# Dosimetric impact of tumor treating field (TTField) transducer arrays onto treatment plans for glioblastomas – a planning study

**DOI:** 10.1186/s13014-018-0976-3

**Published:** 2018-02-23

**Authors:** Christoph Straube, Markus Oechsner, Severin Kampfer, Sophia Scharl, Friederike Schmidt-Graf, Jan J. Wilkens, Stephanie E. Combs

**Affiliations:** 1 Department of Radiation Oncology, Klinikum rechts der Isar, Technical University of Munich (TUM), 81675 Munich, Germany; 2 Deutsches Konsortium für Translationale Krebsforschung (DKTK), Partner Site Munich, Munich, Germany; 3 Department of Neurology, Klinikum rechts der Isar, Technical University of Munich (TUM), Munich, Germany; 4 Department of Radiation Sciences (DRS), Institute for Innovative Radiotherapy (iRT), Helmholtz Zentrum München, Munich, Germany

## Abstract

**Background:**

Tumor-Treating Fields (TTFields) are a novel treatment strategy for glioblastoma (GBM) that is approved for the use concomitantly to adjuvant chemotherapy. Preclinical data suggest a synergistic interaction of TTFields and radiotherapy (RT). However, the dosimetric uncertainties caused by the highly dense arrays have led to caution of applying the TTF setup during RT.

**Methods:**

In a RW3 slab phantom we compared the MV- and kV-CT based planned dose with the measured dose. VMAT-plans were optimized on MV-CTs of an Alderson head phantom without TTF arrays and then re-calculated on the same phantom equipped with TTF arrays. Dose at organs at risk (OAR) and target volumes (PTVs) were compared.

**Results:**

Measurements at a depth of 2, 3 and 4 cm of a RW 3 slab phantom show an attenuation due to TTField arrays of 3.4, 3.7 and 2.7% respectively. This was in-line with calculated attenuations based on MV-CT (1.2, 2.5 and 2.5%) but not with the attenuation expected from kV-CT based calculations (7.1, 8.2 and 8.6%). Consecutive MV-CT based VMAT planning and re-calculation reveals, that the conformity and homogeneity are not affected by the presence of TTField arrays. The dose at organs at risk (OAR) can show increases or decreases by < 0.5 Gy, which should be considered especially in cases next to the scull base.

**Conclusion:**

MV-CT based dose calculation results in reliable dose distributions also in the presence of TTField arrays. There is a small but clinically not relevant interaction between the TTField arrays and VMAT dose application. Thus, daily replacement of TTField arrays is not necessary in regard to deeply located OARs. RT is feasible, when a VMAT treatment plan is optimized to an array free planning CT. As the biologic effect of a concomitant treatment especially on OARs is currently unknown, a concomitant treatment should be performed only within clinical trials.

**Electronic supplementary material:**

The online version of this article (10.1186/s13014-018-0976-3) contains supplementary material, which is available to authorized users.

## Background

Alternating electric fields (tumor-treating fields, TTFields) are a novel treatment strategy for several malignancies, which has shown a stand-alone efficacy comparable to chemotherapeutical agents in recurrent glioblastoma (GBM) [1]. Also in primary GBM a significant benefit for patients treated with TTFields as a concomitant treatment during adjuvant chemotherapy is evident [1–3]. Based on these results, the Optune**©** system (Novocure) gained FDA approval for the treatment of recurrent GBM in 2011 and for the treatment of newly diagnosed GBM in 2015 [4]. In Europe, Optune gained a CE certification in 2015. Furthermore, TTFields were included into the NCCN guidelines for the treatment of primary as well as recurrent GBM as one treatment option [5].

The pathophysiological background of TTFields is still under investigation. However, a prolongation of the cell division, probably elicited by the interference of the alternating electric fields with the condensation of polar tubulin molecules leading to an interruption in spindle formation, seems to be one hallmark of the effect [2, 4, 6, 7]. As a consequence, cells that are exposed to TTFields accumulate within the G2/M-phase; notably, this phase is also associated with a significant vulnerability for radiation induced DNA damages [4, 7]. Besides differences between the proliferation rates of normal tissue cells and tumor cells, TTField effects were described to be frequency dependent [2]. A theoretical synergistic effect of radiation and TTFields was recently corroborated by the results of several preclinical studies, investigating the efficacy of a concomitant treatment with radiation and TTFields in lung cancer and GBM cell lines [6–8]. The increased rate of cell death reported by these articles was explained by an impairment of double-strand break repair, the downregulation of angiogenesis and invasion—related markers such as VEGF and MMP9 also point towards an impaired migration [6, 8]. Furthermore, within the context of chemotherapies, an earlier onset of effective treatments has shown an advantage over use of these therapies within a salvage treatment in low grade glioma as well as in GBM and Medulloblastoma [9–12].

When a synergistic effect of TTFields and radiation seems to be possible, the mode of practically combining these modalities should be investigated. Generally spoken, there are two options of combining a radiotherapy to a TTField treatment. First of all, one could detach the TTField arrays before each fraction and re-attach a new set of arrays afterwards. This strategy would rule out any influence of the TTField arrays onto the dose distribution and an increase of the dose at the organs at risk. On the other hand, a daily change of the arrays could lead to skin irritation and might be associated with longer intervals of de-activated TTFields, with possible negative effects of its efficacy [13]. Additionally, increasing the frequency of array-changes would increase the costs, too. Another strategy would be to leave the arrays attached to the skull during radiotherapy. This strategy could be associated with an increased skin toxicity due to a previously prescribed bolus effect [14]. On the other hand, avoiding to change the arrays on a daily basis could increase the treatment compliance and would possibly reduce the treatment costs. Furthermore, since the TTField transducer arrays consist of materials with a high physical density, there was considerable concern that array application during RT might influence the dose delivery to the patient. Additionally, image artefacts could interfere with the re-positioning of patients. Therefore, before any study concepts with RT and concomitant TTFields are initiated, we focussed to determine any potential interactions between TTField arrays and RT in terms of calculated dose and dose distribution.

## Methods

We first performed a planning study using RW3 water equivalent slabs as a phantom (PTW GmbH, Freiburg, Germany). The phantom was equipped with and without a Novocure Optune TTField array. TTField arrays consist of 9 transducer arrays which each have a thickness of 2.5 mm, a diameter of 2 cm and a physical density of 7.75 g/cm^3^. The electron density (ED) of the arrays as well as the exact chemical composition is unknown. The whole setup was scanned with a Somatom Emotion CT-scanner (kV-CT, 1 mm slice thickness, voxel size 1 × 1 × 1 mm^3^, 130 kV; Siemens Medical Solutions, Erlangen, Germany) and with a TomoTherapy using its MV-CT function (2 mm slice thickness, voxel size 2 × 0.8 × 0.8 mm^3^, 3.5 MV; Accuray Inc., Sunnyvale CA, USA). Irradiation of the RW3 slab phantom was simulated with the treatment planning system (TPS) Eclipse 13.0 (Varian Medical Systems, Palo Alto, CA, USA) using an 0°, 15 × 15 cm^2^, 6 MV photon field with 200 MU. Hounsfield units (HU) were converted to ED based on two different HU-ED conversion tables, them calibrated for the MV-CT and the kV-CT, respectively. We are using kV- as well as MV-CT based treatment planning, the latter one for instance in cases with both sided hip replacements, within the daily clinical routine at our facility. The dose was calculated based on the kV-CT and the MV-CT of the phantom with the AAA13 algorithm. No additional density correction was applied to correct for artefacts or the density of the TTField arrays.

The calculated dose was compared to measurements from irradiating an identical field (15 × 15 cm^2^, 6 MV, 200 MU) to the RW3 slab phantom with and without a TTField array. Measurements were performed on a Trilogy linear accelerator (Varian Medical Systems, Palo Alto, CA, USA). The dose at 2, 3 and 4 cm depth was detected by a 2D diode detector array (MapCheck2, SunNuclear Corp., Melbourne, FL, USA). In all measurements, the TTField device was not connected to a field generator when radiation was applied, as effects of the fields on secondary electrons currently cannot be estimated within any planning system and as a radiation induced damage of the portable field generator cannot be excluded. Therefore, only the passive effect of the TTField arrays can be presented. Each measurement included three technical replicates. The attenuation caused by the TTField arrays was calculated as ratio between the dose without the arrays and the dose with the arrays attached to the uppermost RW3 slab. The attenuated dose was measured within the 71 diodes directly below the TTField arrays and was compared to 71 reference points in the same position of the treatment plans. A difference between measurements and dose calculations of < 3% was deemed to be clinical acceptable.

According to the treatment recommendation of TTFields, the position of the TTField arrays have to be changed every 3 days in order to reduce skin irritations caused by the contact gel below the electrodes. To account for this, the Alderson head-phantom was first scanned with kV-CT and MV-CT without the TTField arrays, and thereafter with four TTField arrays in two different positions (Position “A” and “B”). Each setup included four TTField transducer arrays, with two arrays in opposing positions (i.e. anterior-posterior) while the two remaining arrays were positioned orthogonally (i.e. bi-temporal) (Additional file 1: Figure S1).

Two target volumes were generated to investigate for two distinct questions. PTV1 would have represented a parietal glioblastoma, a location where almost the entire PTV lies within CT slices that are covered by a large number of electrodes. This volume was chosen to investigate whether the electrodes would lead to impairments within the dose distribution to the target volume. PTV2 simulates a temporopolar glioblastoma. Due to its position next to the skull base, irradiation of such volumes often is a trade-off between the dose coverage of the PTV and the dose of the organs at risk, namely the optical nerves, the chiasm and the brainstem. This volume was chosen to investigate, whether the addition of electrodes could lead to an overdosing within the OARs. The effect on the dose distribution caused by the electrodes applied to the head was evaluated by optimizing volumetric arc treatment plans (VMAT) with two arcs with 6 MV for the two PTVs on the MV-CT of the Alderson phantom without electrodes. Subsequently, this plan was copied and recalculated onto MV-CTs of the Alderson phantom with four TTField arrays placed onto the head in position A and B, corresponding to the clinical use of the arrays. The amount of monitor units (MU) was identical for all three plans. The resulting dose distributions were compared according to the ICRU 83 report recommendations by comparing conformity (defined as CI=V95%/PTV), homogeneity (defined as HI = (D_98%_-D_2%_)/D_50%_), D_min_, D_98%_, D_mean_, D_50%_, D_2%_, D_max_ of the PTV as well as according to the D_mean_, D_max_, D_2%_, V_50Gy_ and V_54Gy_ of the OARs [15].

To investigate the impact of the TTField arrays on image quality and registration, we compared kV-CTs, MV-CTs and kV cone beam CTs (CBCT) of the Alderson head phantom with TTField arrays. The latter was acquired using the kV on-board imaging system of the Varian Trilogy.

## Results

### RW3 slab phantom

**Fig. 1:**
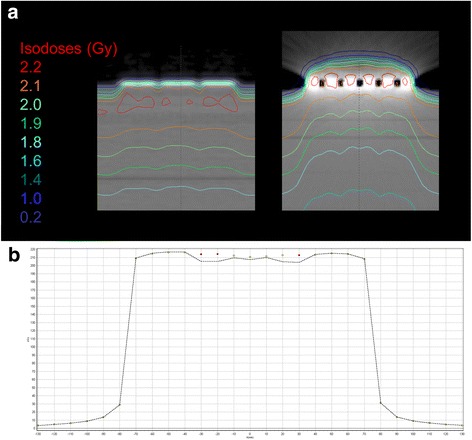
**a** MV-CT (left side) and kV-CT (right side) of a RW3 slab phantom equipped with a NovoCure Optune TTField array planned for a 15 × 15 cm^2^ 0° 6 MV 200 MU photon beam. **b** For verification of the dose calculation, the dose in 2 cm depth was measured in the same setup with and without a TTField array at the surface of a RW3 slab phantom. The dots represent the measurement without the TTField array, the dashed line represents the measurements with TTField

KV-CT based scanning results in significant artefacts within the slices involving the arrays. Due to their high physical density the mean CT-value of the arrays is 3069 HU (SD 1.1), which is limited due to the maximum representable HU value of 3071 HU in our kV-CT scanner. The images were affected by several artefacts, including a feigned thickness of 7 mm and a large halo below and above the arrays onto which a CT-Value of > 1000 HU is projected (Fig. 1a, right panel).

MV-CT-scanning resulted in almost artefact free images (Fig. 1a, left panel). The TTField transducer arrays were represented by a mean density of 2437 HU (SD 134) and were depicted slightly thicker than real (3.6 mm) but with an accurate structure. The difference in thickness, however, can also be caused by the lower z-resolution of the MV-CT.

Comparing the dose calculated on the RW3 slab phantom with and without TTField array to the doses measured in 2, 3 and 4 cm of depth, kV-CT calculation resulted in a dose attenuation by the electrodes of 7.1, 8.2 and 8.6%. The dose calculation based on the MV-CTs resulted in an attenuation of 1.2, 2.5 and 2.5% in 2, 3 and 4 cm, respectively. The dose measurement of the same setup in the same depths with the MapCheck2 diode array resulted in an attenuation of 3.4, 3.7 and 2.7% (Fig. 1b, Table 1). When the doses at 2, 3 and 4 cm of MV-CT based or kV-CT based calculations were compared to the measured doses at the specific depths, this resulted in a difference of 2.2, 1.1 and 0.05% or − 4.2, − 5.0 and − 6.9%, respectively.

**Table 1:** Comparison of the calculated to the measured doses in 2, 3 and 4 cm depth of a RW3 slab phantom. All experiments were performed with a Trilogy linear accelerator irradiating a 6 MV, 15 × 15 cm^2^ field with 200 MU. The doses were measured on 71 points directly below the TTField arrays with a MapCheck2 2D diode detector array. The arithmetic average as well as the standard deviation of the doses at these 71 measure points are presented

	depth	Without TTField arrays		With TTField arrays		Attenuation
		Average (cGy)	StdDev	Average (cGy)	StdDev	
kV	2 cm	214.3	*1.10*	199.1	*4.90*	7.09%
	3 cm	204.6	*0.80*	187.9	*4.90*	8.16%
	4 cm	194.3	*0.60*	177.6	*4.70*	8.59%
MV	2 cm	214.4	*0.80*	211.9	*1.20*	1.17%
	3 cm	204.6	*0.60*	199.5	*1.20*	2.49%
	4 cm	194.8	*0.50*	189.9	*1.20*	2.52%
Phantom	2 cm	214.6	*1.60*	207.4	*4.00*	3.36%
	3 cm	204.9	*1.30*	197.3	*3.40*	3.71%
	4 cm	195.0	*1.10*	189.8	*4.20*	2.67%

A 5 mm area below the electrodes showed an increase in the local dose by 23.7% (D_mean_) and 1.0% (D_max_) for MV-CT and 10.4% (D_mean_) and 3.3% (D_max_) for kV-CT, respectively, indicating a bolus effect of the electrodes. This changes could not be verified with the MapCheck2 system, as the minimum depth for measurements with this system is 2 cm.

### Alderson head phantom

**Fig. 2:**
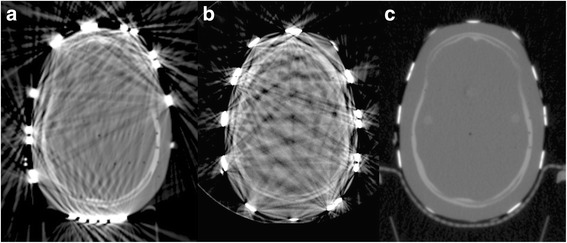
kV-CT (**a**), CBCT (**b**) and MV-CT (**c**) of an Alderson head phantom equipped with TTField transducer arrays (Range − 1000 to 3071 HU). In kV-CT and CBCT, bony structures are only visible when they are not within the direct vicinity of TTField arrays and streaky artefacts are present. In contrast, MV-CT is almost free of artefacts, all bony structures are clearly visible and also structures with a low radiographic density, i.e. bore holes, are resolved.

As expected from the results from the RW3 slab phantom scans, kV-CTs and CBCTs of the head phantom suffered from a large burden of artefacts in the slices that involved electrodes (Fig. 2). Due to the higher photon energy there were almost no artefacts present in the MV-CT. Bony structures from the scull base were clearly visible in all modalities. When the registration was based on the bony structures of the phantom, including the skull base, registration of the images was not affected by the presence of the TTField arrays.

**Fig. 3:**
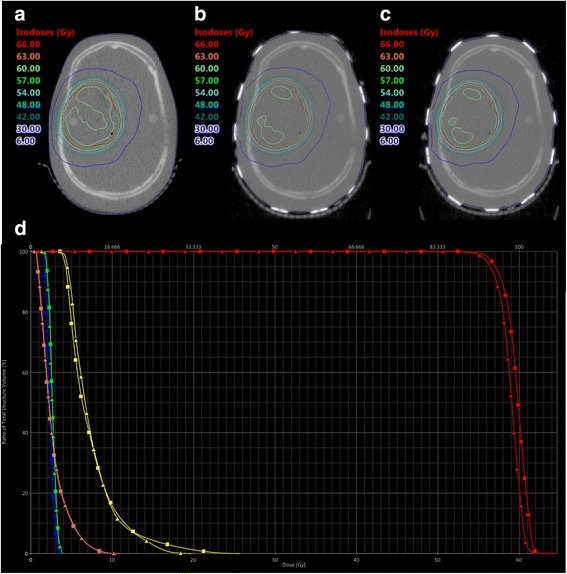
MV-CTs of an Alderson head phantom without (**a**) and with TTField arrays in position A (**b**) and B (**c**). A treatment volume resembling the PTV for a parietal glioblastoma was optimized to the MV-CT in (**a**) and re-calculated to MV-CT (**b**) and (**c**). (**d**) shows a DVH-comparison between the plan for (**a**) (squares) and (**b**) (triangles). Red: PTV, yellow: chiasm, blue: left optical nerve, green: right optical nerve, pink: brainstem

**Fig. 4:**
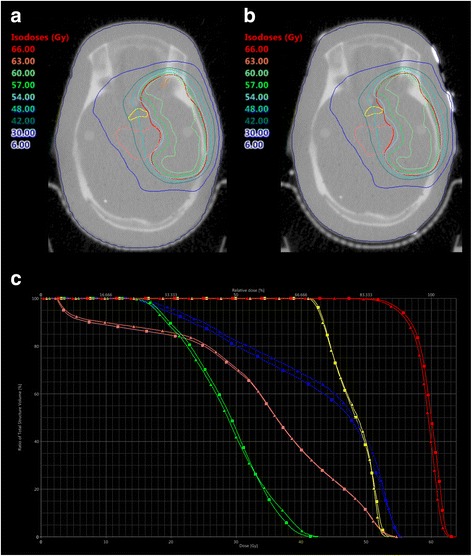
MV-CTs of an Alderson head phantom without (**a**) and with TTField arrays (**b**). A treatment plan for a target volume resembling the PTV for a temporopolar glioblastoma was optimized on the MV-CT in (**a**) and re-calculated to MV-CT (**b**). **c** shows a DVH-comparison between the plan for (**a**) (squares) and (**b**) (triangles). Red: PTV, yellow: chiasm, blue: left optical nerve, green: right optical nerve, pink: brainstem

In both PTVs, the addition of TTField arrays led to a negligible reduction of the conformity and minor changes of the mean and maximum dose up to 1.0 Gy (Figs. 3 and 4, Tables 2 and 3). In the presence of TTField arrays, D_min_ was up to 4.4 Gy lower as compared to plans without TTField arrays. The volume compromised by this decrease had a size of 0.05 cm^3^. No changes in the homogeneity of the dose distribution were found. The OARs, namely the optical nerves, the chiasm and the brainstem, were mostly stable or decreased (Tables 2 and 3). The largest increase was observed at the optic nerves, where an increase of up to 0.4 Gy was present.

**Table 2:** Dose distribution and conformity on PTV1, resembling a target volume for a parietal glioblastoma

PTV1	Optimized Plan	Re-Calculation on Position A	Re-Calculation on Position B
PTV			
D_max_	64.6 Gy	64.2 Gy	63.7 Gy
D_2%_	61.7 Gy	61.0 Gy	60.8 Gy
D_mean_	59.7 Gy	58.9 Gy	58.7 Gy
D_50%_	59.9 Gy	59.1 Gy	58.8 Gy
D_98%_	56.2 Gy	55.6 Gy	55.3 Gy
D_min_	50.2 Gy	45.8 Gy	47.2 Gy
Conformity (V_95%_/PTV)	0.93	0.89	0.87
Homogenity ((D_2%_-D_98%_)/D_50%_)	0.09	0.09	0.09
Chiasm			
D_max_	25.7 Gy	19.8 Gy	20.7 Gy
D_2%_	18.4 Gy	15.6 Gy	16.3 Gy
D_mean_	7.4 Gy	7.4 Gy	7.7 Gy
V_54Gy_	0 cm^3^	0 cm^3^	0 cm^3^
V_50Gy_	0 cm^3^	0 cm^3^	0 cm^3^
Left Optical Nerve			
D_max_	3.4 Gy	3.8 Gy	3.8 Gy
D_2%_	3.2 Gy	3.5 Gy	3.6 Gy
D_mean_	2.3 Gy	2.4 Gy	2.4 Gy
V_54Gy_	0 cm^3^	0 cm^3^	0 cm^3^
V_50Gy_	0 cm^3^	0 cm^3^	0 cm^3^
Right Optical Nerve			
D_max_	3.6 Gy	3,9 Gy	3.9 Gy
D_2%_	3.5 Gy	3.6 Gy	3.7 Gy
D_mean_	2.7 Gy	2.7 Gy	2.7 Gy
V_54Gy_	0 cm^3^	0 cm^3^	0 cm^3^
V_50Gy_	0 cm^3^	0 cm^3^	0 cm^2^
Brainstem			
D_max_	11.0 Gy	10.7 Gy	11.2 Gy
D_2%_	7.5 Gy	7.5 Gy	7.7 Gy
D_mean_	2.7 Gy	2.7 Gy	2.7 Gy
V_54Gy_	0 cm^3^	0 cm^3^	0 cm^3^
V_50Gy_	0 cm^3^	0 cm^3^	0 cm^3^

**Table 3:** Dose distribution and conformity on PTV2, resembling a target volume for a temporopolar glioblastoma

PTV2	Optimized Plan	Re-Calculation on Position A	Re-Calculation on Position B
PTV			
D_max_	63.8 Gy	63.8 Gy	63.3 Gy
D_2%_	62.4 Gy	62.1 Gy	61.9 Gy
D_mean_	59.6 Gy	59.1 Gy	59.0 Gy
D_50%_	60.0 Gy	59.5 Gy	59.4 Gy
D_98%_	53.5 Gy	52.9 Gy	53.1 Gy
D_min_	45.4 Gy	42.4 Gy	45.9 Gy
Conformity (V_95%_/PTV)	0.89	0.86	0.86
Homogenity ((D_2%_-D_98%_)/D_50%_)	0.15	0.15	0.15
Chiasm			
D_max_	54.5 Gy	53.5 Gy	53.3 Gy
D_2%_	52.7 Gy	52.3 Gy	52.4 Gy
D_mean_	48.0 Gy	48.0 Gy	47.6 Gy
V_54Gy_	< 0.01 cm^3^	0 cm^3^	0 cm^3^
V_50Gy_	0.29 cm^3^	0.27 cm^3^	0.24 cm^3^
Left Optical Nerve			
D_max_	55.5 Gy	55.7 Gy	55.4 Gy
D_2%_	54.7 Gy	54.7 Gy	54.6 Gy
D_mean_	42.3 Gy	43.3 Gy	41.7 Gy
V_54Gy_	0.02 cm^3^	0.02 cm^3^	0.02 cm^3^
V_50Gy_	0.11 cm^3^	0.12 cm^3^	0.13 cm^3^
Right Optical Nerve			
D_max_	42.6 Gy	42.7 Gy	43.0 Gy
D_2%_	39.3 Gy	40.4 Gy	39.9 Gy
D_mean_	28.6 Gy	28.5 Gy	27.4 Gy
V_54Gy_	0 cm^3^	0 cm^3^	0 cm^3^
V_50Gy_	0 cm^3^	0 cm^3^	0 cm^3^
Brainstem			
D_max_	55.5 Gy	55.7 Gy	55.1 Gy
D_2%_	52.7 Gy	52.6 Gy	52.2 Gy
D_mean_	33.9 Gy	34.3 Gy	33.7 Gy
V_54Gy_	0.09 cm^3^	0.10 cm^3^	0.02 cm^3^
V_50Gy_	2.82 cm^3^	2.78 cm^3^	2.48 cm^3^

## Discussion

In the present work, we used MV-CT based treatment planning and re-planning to investigate changes in the dose delivery introduced by high density TTField arrays. This information is prerequisite for establishing study protocols that can investigate the safety and efficacy of a concomitant use of TTFields during a course of radiotherapy.

We used the AAA13 algorithm to calculate the dose distributions. This algorithm is based on electron densities which previously have to be converted from the HU values of kV or MV-CTs. This is done by conversion tables that correlate HU values and electron densities [16]. The conversion tables are derived from scans of different materials with known electron densities and are machine-specific. We use distinct conversion tables for MV-CT based dose calculations within our daily routine, especially when patients with hip-replacements have to be treated within the pelvic area.

The presence of high density materials, such as TTField arrays with a physical density of 7.75 g/cm^3^, can lead to photon starvation that propagate to streak artefacts [17]. These artefacts are consequently represented by apparently high, in most cases maximum, HU values. In our case, the arrays as well as parts of the halo were represented by up to 3071 HU with kV-CT, corresponding to a relative electron density of 2.52. This value is lower than expected from the physical density, however, the true ED of the arrays is unknown. Furthermore, due to the artefacts, the thickness of the arrays is seemingly bigger (7 mm in kV-CT as compared to 2.5 mm of physical thickness). Altogether, artefacts in general and also in our special case lead to an impaired accuracy of the dose calculation [18]. This effect can be impressively seen in case of the kV-CT based dose calculation in Fig. 1a.

CT images that are based on higher energy photons, such as our MV-CTs with 3.5 MV, are less prone by artefacts. This is due to the energy dependence of different mechanisms of photon absorption, namely the photoelectric effect or the Compton effect. As a consequence, scanning by a MV-CT results in an almost artefact free image - also in the presence of TTField arrays. A second aspect is the finding, that the allocated CT number of material with a high physical and electron density inversely correlates with the photon energy used for CT-scanning [19]. This leads to shallower ED-HU-conversion curve. The HU-value of the high density arrays is therefore still within the range of the HU table and not at its edge (2854 HU in parts of the arrays, representing a relative ED of 3.9). Based on these considerations we assumed that a MV-CT based dose calculation is more reliable in presence of TTField arrays than a dose calculation based on kV-CTs. The general applicability of MV-CTs for radiotherapy planning was already shown by other groups [20]. This assumption was further substantiated by measurements with a linear accelerator which showed a good accordance of the measurements with the calculations in terms of depth doses as well as with attenuations caused by the TTField arrays.

In principle, the accuracy of a kV-CT based dose calculation can theoretically be improved by extending the HU-table or replacing the electrodes in the CT dataset by the true electron density – which unfortunately is unknown. However, image artefacts outside the electrodes must also be corrected by the real tissue density and a replacement with one density might be insufficient, too. In addition, finding the correct position of the electrodes can be hampered by the artefacts. In contrast to this approach, MV-CT based dose calculation is free of additional corrections.

Based on these considerations, we investigated the impact of TTField arrays on the MV-CT based calculated dose delivery in two hypothetic cases of glioblastoma. Comparison of the plans for two exemplary target volumes showed only minor effects on the plan quality. The homogeneity index was not affected when the TTField arrays were added to the setup and the conformity index showed only minor differences. Therefore, radiotherapy with simultaneously applied TTField arrays seems to be possible without negatively affecting the tumor control probability as similar doses are delivered independently from the presence of the arrays. Importantly, to our knowledge, there are currently no data available about possible synergistic effects between TTFields and radiation onto normal tissues.

The doses at deeply seated OARs, such as the chiasm, the optic nerves or the brainstem, were also not increased by more than 0.4 Gy by the presence of the arrays. This is within the range of the inherent dose calculation uncertainties of current treatment planning systems and largely below uncertainties due to positioning errors [21, 22]. However, even a small increase in the D_max_ could have detrimental late effects, the dose constraints should be chosen conservatively and met exactly, especially as additional biologic TTField effects on normal tissues are unknown. In summary, based on the criteria of ICRU report 83, the absorbed doses within the PTV as well as the OAR are affected by the presence of TTField arrays, but the differences are not likely to be of clinical relevance [15]. Or with other words: if an increased rate of adverse events would be observed during concomitant use of TTFields and radiotherapy, with TTField arrays attached during irradiation, then this likely would not be due to a change within the dose delivery but to an additional biologic effects.

Besides only modest effects on the dose-distribution within the depth, a bolus effect caused by the electrodes was shown by us as well as by Bender et al. [14]. Notably, the bolus effect to the skin was not the main focus of this manuscript and was therefore calculated only for static 0° fields. The overdosing within the skin can still be relevant, but as the positions of the arrays are changed every 3 days, potential side effects, mostly to the skin, can be attenuated. However, since hair loss is present depending on the size and the location of the lesion of the GBM, such overdosing at the skin and hair level might increase the rate of permanent alopecia and should be kept in mind when counselling the patients.

Additionally, daily repositioning can be performed precisely even with the TTField system present during the treatment process, especially when MV-CT can be used for positioning. As the OAR of the CNS are mostly located at the skull base, and as this area is usually not covered by the TTField arrays, sufficient registration of the skull base is also possible with kV-CT or CBCT, too. When soft tissue information is deemed to be important for repositioning, MV-CT based imaging or daily change of the TTField arrays can be performed.

Notably, the biology effects of TTFields and potential additive effects with radiation have only been studied in cell lines of malignant tumors so far and no preclinical evidence about the effect of a concomitant application of TTFields and RT on normal tissue exist. There is a potential risk, that a combination of the two modalities might also increase the risk of side effects. For instance, a reduced fractionation effect could be assumed based on a delayed repair of radiation induced DNA damages, even though this was only shown for malignant cells [8]. This potentially could also lead to an increase in severe late effects, such as radionecrosis. On the other hand, as there is only limited proliferative activity within healthy adult brain tissue and no severe neurotoxicity after TTField-treatment has been reported so far, one could also assume a relatively low risk for an excessive increase in normal tissue side effects by combining these two modalities. Therefore, further preclinical studies on the impact of this modality in normal tissue radiation tolerance as well as treatment within clinical trials are highly recommended before the routine use of concomitant radio-TTField-treatments.

## Conclusion

Electrode arrays of the Optune TTField-system cause streak artefacts on kV-CTs which are impairing trustworthy dose calculations. Our data show that application of the TTField arrays during treatment planning and application of RT does not impact dose calculations, if the dose is calculated based on a MV-CT dataset. Use of MV-CTs for optimizing VMAT plans as well as for recalculation of already optimized VMAT treatment plans reveal only minor effects of TTField arrays on the conformity, homogeneity and median dose as well as the doses at OARs. Noteworthy, an increased skin toxicity seems to be likely. As the area above the skull base is broadly spared by the TTField arrays and consequently is only slightly effected by artefacts, repositioning can be safely performed also with CBCTs. Based on these findings, irradiation of VMAT plans optimized without the presence of TTField arrays seems to be feasible, yet a possible adverse synergistic interaction of TTFields and radiation onto normal tissues should be considered, too.

## Additional file

Additional file 1: **Figure S1**: Example of TTField arrays attached to an Alderson head phantom. (PNG 25896 kb)

## Abbreviations

AAA: Anisotropic analytical algorithm
CBCT: Cone beam computed tomography
CT: Computed tomography
FDA: Food and drug association
GBM: Glioblastoma
GTR: Gross total resection
GTV: Gross tumor volume
HU: Hounsfield units
ICRU: International commission on radiation units
kV-CT: Kilo voltage computed tomography
MU: Monitor units
MV-CT: Mega voltage computed tomography
NCCN: National comprehensive cancer network
OAR: Organ at risk
PTV: Planning target volume
RT: Radiotherapy
SD: Standard deviation
TTField: Tumor treatment fields
VMAT: Volumetric arc therapy
