# Supplementary figures and images for: Dosimetric impact of tumor treating field (TTField) transducer arrays onto treatment plans for glioblastomas – a planning study
Source: Radiat Oncol. 2018 Feb 23;13:31. doi: 10.1186/s13014-018-0976-3 (PMC5824562; doi:10.1186/s13014-018-0976-3)

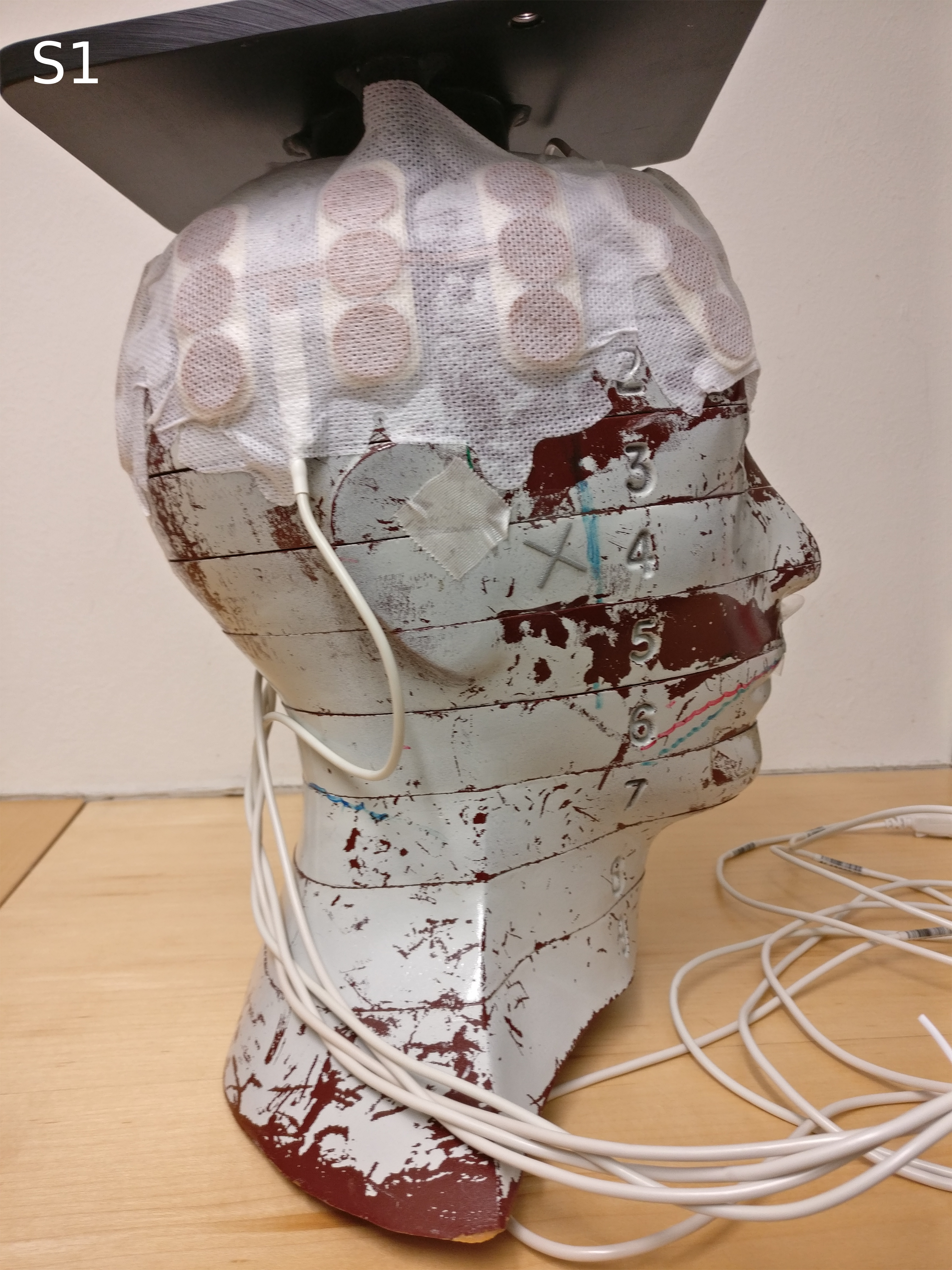

Supplement: Supplementary file 1 — Figure S1: Example of TTField arrays attached to an Alderson head phantom. (PNG 25896 kb) [file 13014_2018_976_MOESM1_ESM.png]
